# Premotor-Motor Interhemispheric Inhibition Is Released during Movement Initiation in Older but Not Young Adults

**DOI:** 10.1371/journal.pone.0052573

**Published:** 2012-12-19

**Authors:** Mark R. Hinder, Hakuei Fujiyama, Jeffery J. Summers

**Affiliations:** Motor Control Laboratory, School of Psychology, University of Tasmania, Tasmania, Australia; Weill Cornell Medical College, United States of America

## Abstract

Neural interactions between contralateral motor regions are thought to be instrumental in the successful preparation, and execution, of volitional movements. Here we investigated whether healthy ageing is associated with a change in functional connectivity, as indicated by the ability to modulate interhemispheric interactions during movement preparation in a manner that assists rapid movement responses. Thirteen young (mean age 22.2 years) and thirteen older (68.5 years) adults rapidly abducted their left index finger as soon as possible in response to a visual imperative signal, presented 500 ms after a visual warning signal.

Interactions between left dorsal premotor cortex (LPMd) and right primary motor cortex (RM1) and between left primary motor cortex (LM1) and RM1 were investigated at six time points between the warning signal and the volitional response using paired-pulse transcranial magnetic stimulation. Relative to the inhibitory interactions measured at rest, both young and older adults released LM1-RM1 inhibition beginning 250 ms after the warning signal, with no significant differences between groups. LPMd-RM1 interactions became facilitatory (from the onset of the imperative signal onwards) in the older, but not the young, group. Regression analyses revealed that for the older adults, modulation of LPMd-RM1 interactions early in the preparation period was associated with faster responses, suggesting that specifically timed modulation of these pathways may be a compensatory mechanism to offset, at least in part, slowing of motor responses. The results suggest a greater reliance on premotor regions during the preparation of simple motor actions with advancing age.

## Introduction

Successful execution of many sensory and motor tasks relies upon complex interhemispheric communication that occurs via fibres of the corpus callosum [1]. Of particular interest with respect to movement control is the interhemispheric interaction (IHI) between the two primary motor cortices (M1). These interactions have been extensively studied using paired pulse transcranial magnetic stimulation [2] (TMS) at rest [2], [3] and during movement preparation [4]–[6]. During preparation and execution of a task undertaken with the right hand, IHI from the passive (right) to active (left) cortex is reduced to ‘release’ the planned action, while IHI onto the non-responding cortex is increased to prevent unwanted mirror activity [4], [7] suggesting that modulation of IHI plays a functional role in movement control.

Direct interhemispheric pathways between primary motor regions are relatively sparse compared to the more dense interhemispheric pathways upstream of primary motor cortex [8] (for a review see [9]). Accordingly, task-specific alterations in transcallosal interactions from premotor regions [9] may also play an important role in movement control. Such interactions would be hypothesised to be particularly important during movement preparation given that ‘preparatory neurons’ are more abundant in premotor, compared to motor, regions during a choice reaction time task [10]. Specifically, the left (L) PMd assumes a dominant role [11]–[13] in the preparation of movements of either hand, when movements occur in response to external cues. At rest, the interactions between PMd and the contralateral M1 appear to be dependent upon the intensity of the conditioning pulse [14], [15], with low intensity conditioning pulses (60–80% active motor threshold) generally leading to interhemispheric facilitation and higher intensity pulses (>110% resting motor threshold) eliciting interhemispheric inhibition similar to that observed between contralateral M1s. Given the dominant role of LPMd during preparation of movements, a number of studies have investigated modulation of LPMd to right (R) M1 pathways in the period when participants prepare to move the left or right hand as quickly as possible in response to external cues [16], [17]. LPMd-RM1 interactions were inhibitory at rest, but became facilitatory during the preparation of right hand actions, while the resting state inhibition was maintained during the preparation of left hand actions.

While pioneering work suggested that, given a conditioning pulse of appropriate intensity, IHI between M1s could be elicited with an interval between the conditioning and test stimulus of between 10 and ∼40–50 ms [2], [18], the majority of research subsequently adopted 10 ms ISI for testing interactions between contralateral motor areas [4], [19]–[21]. However, a growing body of evidence suggests that rather than being present at *all* ISIs from 10 ms to 40 ms IHI is observed at two distinct ISIs, of around 10 and 40 ms [22], [23]. Moreover, while these two ‘phases’ of IHI – referred to as short IHI (SIHI) and long IHI (LIHI) – share several characteristics in terms of modulation during various tasks, they appear to be mediated by different physiological mechanisms [22], [24], [25] and may be differently affected by healthy ageing [26]. It has been suggested that LIHI is mediated by postsynaptic GABA_B_ receptors [22], [23]; however, the mechanisms mediating SIHI are still unclear. Ni et al. [25] recently reported that SIHI and LIHI were present between a number of distinct motor regions in the left hemisphere, including PMd and M1, and the contralateral (right) M1.

Despite a growing literature indicating that the nature of the interactions between the pre-motor or motor cortex in one hemisphere and the contralateral motor cortex can be modulated during task execution and preparation, extant studies have not been specifically designed to assess how these neurophysiological measures correlate with specific attributes of behaviour. In a recent attempt to link physiological function with task performance, Liuzzi et al. [6] assessed task-related changes in interhemispheric interactions during the preparation of a simple reaction time task, and correlated these changes with the ability to execute more complex bimanual and unimanual tapping rhythms - for a brief review, see [27]. SIHI was assessed between RPMd and LM1, and between RM1 and LM1 during the preparation of right hand movements. More facilitatory RPMd-LM1 interactions during the early part of the preparation period were associated with better performance in a bimanual coordination pattern requiring asynchronous activation of homologous muscles in contralateral limbs. In contrast, reduced inhibition between primary motor regions (RM1-LM1) was associated with better performance in a bimanual tapping task requiring simultaneous activation of homologous muscles. It therefore appears that an ability to control the nature of interhemispheric interactions between distinct motor regions may be associated to specific aspects of bimanual motor coordination.

Degradation of the corpus callosum that can occur with advancing age may be linked to behavioural observations that older adults exhibit bilateral cortical activity [28] and bilateral muscle activity [29]–[31] during actions which are intended to be unilateral. Indeed, it appears that those changes that occur in the brain as a result of normal ageing may result in reduced capability to modulate some [26] but not all [7], [26] interhemispheric inhibitory mechanisms during motor tasks undertaken with the upper limbs, which may impact on the ability to execute certain dextrous motor actions. Specifically, task-related modulation of M1-M1 SIHI seems to be unaffected by age [7], [26], while modulation of M1-M1 LIHI appears to exhibit an age-related decline [26]. To date, studies have not addressed whether healthy ageing is associated with changes in SIHI or LIHI between PMd and the contralateral M1. However, a recent functional magnetic resonance imaging study [28] found that during a left-hand force production task, task-related activity in the left and right PMd was positively correlated with participants' age. Consistent with this finding, a number of other studies [32]–[34] suggest that during interlimb coordination older adults exhibit greater activation in frontal and pre-frontal brain regions than young adults. Taken together, these imaging and electroencephalography studies suggest that premotor areas play a greater role during motor tasks for older, compared to young, adults. Accordingly, any breakdown of interhemispheric interactions emanating from premotor areas that occur with advancing age would assume significance for motor control during later life.

The present study, therefore, was designed to investigate the modulation of transcallosal interactions between the LM1 and the RM1, and between the LPMd and the RM1 during preparation of a simple motor task. In groups of young and older participants we assessed SIHI and LIHI mechanisms [22], [23], [25] and hypothesised that due to the dominant role of LPMd in movement preparation [11]–[13], functional connectivity (i.e., modulation of the interhemispheric interaction) between LPMd and RM1 would play an important role in permitting fast motor responses, especially in older adults.

## Methods

### Participants

Thirteen young (mean age ±95% confidence intervals (CI) 22.2±2.4 years) and thirteen older (68.5±2.9 years) adults volunteered to take part in the study. All participants were right handed according to the Edinburgh Inventory [35], were free from neurological deficits and had normal or corrected-to-normal vision. Participants signed an informed consent form prior to participating in the experiment, which had been approved by the UTAS institutional ethics committee.

### Movement task

The experiment was designed to assess interhemispheric interactions during a simple reaction time task. Participants were seated comfortably and placed their forearms on a horizontal board mounted on a table. The palms faced down and the elbows were bent at approximately 120°. The hands were restrained using vertical pegs inserted into the board [36]. These restraints were designed to restrict movements to the second metacarpo-phalangeal joint [37], [38] and helped to maintain a consistent posture (with forearm muscles relaxed) throughout the experiment.

A vertical array of light emitting diodes (LEDs) mounted within a black box was placed at eye level approximately 80 cm in front of participants. The upper orange LED was illuminated for 500 ms and acted as a warning signal (WS), after which the lower green LED was illuminated for 500 ms and acted as the imperative (‘go’) signal (IS). Participants were required to respond as quickly as possible to the IS by rapidly abducting their left index finger in the horizontal plane. They were instructed to move in the horizontal plane by skimming across the surface of the low-friction board and asked to isolate the movements to the second metacarpo-phalangeal joint of the index finger [36]. A short (500 ms) warning signal period was used to promote the preparation of actions as much as possible [39], [40], which we envisaged would be evident as changes in interhemispheric interactions during the preparation period.

Two blocks of 24 trials were initially undertaken in the absence of any TMS. These blocks served to provide practice for the participants and also provided an initial measure of response times to determine TMS timing in subsequent stimulation trials. Three trials in each of these blocks were ‘catch’ trials, in which the WS was not followed by the IS. In these trials participants were required *not* to respond, i.e., not abduct their index finger. By including catch trials, we ensured that while the WS signalled the impending IS, the WS itself could not be used to initiate a response.

Two subsequent blocks (of 36 trials) were conducted in which interhemispheric interactions were assessed at rest (i.e., no motor task was undertaken). One of these blocks assessed interactions between LM1 and RM1 and one block investigated interactions between LPMd and RM1; the order of these blocks was counterbalanced across participants (see *Transcranial Magnetic Stimulation* section, below, for details on stimulation parameters).

The main part of the experiment consisted of twelve blocks of 36 trials in which interhemispheric interactions were assessed during movement preparation. Six blocks investigated LM1-RM1 interactions and six blocks investigated LPMd-RM1 interactions; the order of blocks was counterbalanced across participants. Thirty of the 36 trials in each block were warned ‘go’ trials in which TMS was applied at various points between the WS and IS and between the IS and the onset of the volitional muscle activity (see *Transcranial Magnetic Stimulation* section). Three trials were warned ‘go’ trials in the absence of TMS; these trials permitted us to track response speeds in the absence of TMS across the experiment. The remaining three trials in each block were catch trials (with no TMS) to circumvent the early release of actions in response to the WS. The inter-trial interval was 5–7 s, such that after completion of each finger movement there was ∼3.5–5.5 sec before the subsequent warning signal was presented. Participants were permitted to rest between blocks, if desired. The experimental procedure including set-up, lasted no more than two hours.

### Electromyographic recording

Movement related muscle activity and motor evoked potentials (MEPs) were recorded from the left first dorsal interosseus (FDI), the muscle primarily responsible for execution of the volitional movement, and from its homologue in the non-responding right hand. Data were stored on a computer for offline analysis.

### Transcranial magnetic stimulation

We used paired pulse TMS [2] to investigate the interactions from the left to the right hemisphere. We chose this ‘direction’ of interaction (i.e., left to right) on the basis that, in simple reaction time tasks, LPMd appears to plays a dominant role in the preparation of movements undertaken by either hand [10]–[13] whereas the RPMd plays a more pivotal role in bimanual coordination [6], [41]. TMS was delivered to the left (‘conditioned’) and right (‘test’) cortices using two Magstim 200 units (Magstim Company, Dyfed, UK) and two ‘branding iron’ style figure of eight coils (with an outside diameter of ∼50 mm for each wing). Branding iron coils were chosen as one coil could be placed on each cortex without compromising either coil's positioning relative to the respective motor hotspots (see below). One experimenter was responsible for maintaining the specific scalp position of each coil. Optimal coil positions for eliciting MEPs from the left and right FDI (with posterior to anterior current direction, i.e., coils at ∼45 degrees to the midline) were determined prior to the experimental trials, and marked on the scalp. Resting motor thresholds (RMT), from which the stimulation intensities were derived, were determined as the minimum intensities required to elicit MEPs of peak-peak amplitude >50 μV (in the period 20–80 ms following TMS stimulation) in the right and left FDI muscles in 3 out of 5 consecutive trials when stimulating at the pre-determined hotspots [36], [42]–[44].

During the interhemispheric interaction trials conducted at rest and at each time point during the movement preparation trials, we applied three different types of stimulation. One third of the TMS trials involved a single ‘test’ stimulus (TS) applied to the right cortex at the motor hotspot for the left FDI muscle at 130% left FDI RMT. These trials enabled the excitability of the corticospinal pathways to the left FDI to be determined. In the other TMS trials in each block a conditioning pulse (CS at 110% RMT; [45]) was delivered to either the motor hotspot for the right FDI muscle (i.e., LM1) or to LPMd prior to the test pulse to determine the nature of the interaction of that area onto the right primary motor cortex [2]. The location of the LPMd was determined as 8% of the nasion-inion distance anterior to the left FDI representation within primary motor cortex [45]. For left M1 conditioning, the interstimulus interval between the CS and the TS was either 10 or 40 ms, which allowed assessment of SIHI and LIHI, respectively [22], [25], [46]. In the case of LPMd conditioning, 8 and 40 ms ISIs were chosen to assess SIHI and LIHI. We note that 8 ms, rather than a 10 ms ISI, was used to assess PMd-M1 SIHI as pilot testing revealed a somewhat more robust inhibitory effect at this ISI – a finding consistent with the 6–8 ms used in previous research [15], [45].

**Figure 1:**
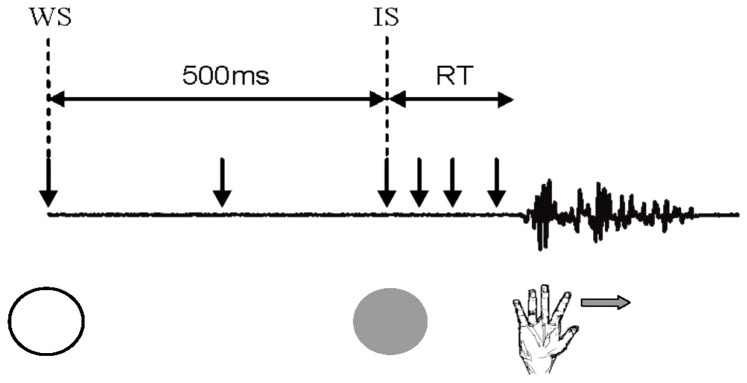
Experimental task and TMS timing. The warning signal (orange light, WS, here represented by the white circle) was presented for 500 ms followed by the green imperative (‘go’) signal (IS), represented by the gray circle. Participants responded to the IS as quickly as possible by rapidly abducting their left index finger in the horizontal plane. TMS was delivered at six time points as indicated by the vertical arrows.

In the two blocks in which TMS was applied at rest, 12 single pulse, and 12 paired pulse trials at each ISI (total of 36 TMS trials per block) were administered for each interhemispheric pathway (i.e., LM1-RM1 and LPMd-RM1 in different blocks). Across the 12 movement preparation blocks, we administered 12 single pulse and 12 paired pulse trials at each ISI for each pathway at six time points prior to onset of volitional response (total of 360 TMS trials). Note that because 6 of the 36 trials in each block were non-TMS trials to circumvent early responses and to track response times in the absence of TMS this gave rise to a total of 12 blocks of 36 trials (432 trials). In movement preparation blocks TMS was applied coincident with the onset of the warning signal, 250 ms after onset of the warning signal, coincident with the IS and at three further time points established on an individual participant basis, as determined by mean response times (figure 1- also see *Data Analysis* section for determination of response times) in the second of the two practice blocks. Specifically, TMS was applied at a delay (with respect to the IS) equivalent to 25, 50 and 80% of each individual's response time.

### Data analysis

Participants' response times (RTs) were determined in the two initial blocks in which all trials were conducted without TMS, and in the three trials of each experimental block in which TMS was not applied. Accordingly, we derived baseline response times, and tracked response times throughout the experiment. Response time was determined as the interval between presentation of the imperative signal and onset of muscle activity in the left FDI, defined as the time at which root mean square (rms) EMG first increased above a threshold level equivalent to 4 times background EMG determined prior to presentation of the warning signal. RTs reported here are therefore comparable to ‘premotor time’ as reported in some studies.

In TMS stimulation trials, any trial in which rms EMG exceeded 0.025 mV in a 40 ms time window immediately prior to TMS stimulation was excluded from MEP analysis. Corticospinal excitability was determined as the average peak-peak MEP in the left FDI muscle in a time window 20–80 ms following stimulation in single pulse stimulation trials. Corticospinal excitability at each time point during movement preparation trials was normalised to MEP amplitudes in response to single pulses at rest to yield normalised MEP (nMEP). nMEP values greater than 1 indicate facilitation (increased excitability), while values less than 1 indicate suppression (reduced excitability) relative to rest. Interhemispheric interactions (LM1-RM1, LPMd-RM1) at each ISI (representing SIHI and LIHI) were determined as the average MEP amplitude (determined as described above) following paired stimulation, relative to the average MEP amplitude in response to single pulse TMS (i.e., ratio). These ratios are referred to as IHI; values greater than 1 represent a facilitatory interaction, while values less than 1 represent inhibitory interactions. SIHI and LIHI ratios at each time point during movement preparation were subsequently normalised to the comparable IHI ratio determined at rest and are referred to as nIHI [7]. nIHI values greater than 1 represent facilitatory changes during movement preparation while values less than 1 represent inhibitory changes during movement (relative to rest). Normalisation of MEP amplitudes and IHI ratios enabled fully factorial ANOVAs to be conducted, and precludes the data from being biased by any particular participant with particular high/low values at rest.

Between group comparisons of RT, RMTs and corticospinal excitability at rest were undertaken using independent samples *t*-tests. ANOVA was undertaken to assess nMEP with time as a within-subjects factor and age as a between-subjects factor. IHI (at rest) and nIHI (movement preparation) were compared using ANOVA with the factors ISI and age (IHI) and time, ISI and age (nIHI) for each pathway (LM1-RM1, LPMd-RM1). To determine whether any modulation of the interhemispheric interactions observed during movement preparation was associated with task performance, multiple regressions were undertaken. Following previously reported techniques [6], the extent of the IHI modulation at each time point was entered as independent variables (predictors) with equal weighting (using a ‘forward’ enter method, with F probabilities of 0.05 and 0.10 used as the inclusion and exclusion criteria, respectively). RT was the dependent variable. Separate regressions were undertaking for each age group, for PMd-M1 and M1-M1 pathways, and each ISI.

## Results

### Behavioural task

The task was well executed by all participants. For the young adults an average of 3.7% of trials (15 of 396 trials) were rejected due to volitional bursts of muscle activity being recorded prior to the IS or levels of background rms EMG (prior to TMS stimulation) above 0.025 µV. The rejection rate was only 2.0% (8 of 396 trials) for older adults. Furthermore, none of the participants executed many undesired motor responses on the catch trials. Indeed, the average number of ‘false go’ movements in the three catch trials of each block was 0.35±0.18 and 0.22±0.15 for the young and older groups, respectively; these values did not differ significantly between participant groups (Independent samples *t*-test *t*_24_ = 1.18, p = 0.249) and did not vary substantially across experimental blocks.

Young adults exhibited mean RTs that were significantly faster than the older group (Mean ±95% CIs were 190±26 ms (young) and 236±26 ms (older); Independent samples *t*-test *t*_24_ = 10.76, p<0.001). For both groups, reaction times varied little in the non-TMS trials across each block of the experiment (95% CIs across blocks: 7.6 ms, 4.3 ms for the older and young groups, respectively), suggesting that any task-related adaptation or fatigue – if present – had a negligible influence upon response times.

### TMS parameters

Independent samples *t*-tests revealed that resting motor threshold (RMT), expressed as a percentage of maximum stimulator output, for the left (young: 44.3±3.0%; older: 47.7±4.6%) and right (young: 45.0±3.1%; older: 49.2±3.9%) FDI muscle did not vary significantly between groups (L FDI: *t*_24 = _1.20, p = 0.241; R FDI *t*_24 = _1.67, p = 0.107), nor did RMT vary between each hand for each group (young: *t*_24 = _0.32, p = 0.755; older: *t*_24_  = 0.50, p = 0.623).

### Corticospinal excitability

**Figure 2:**
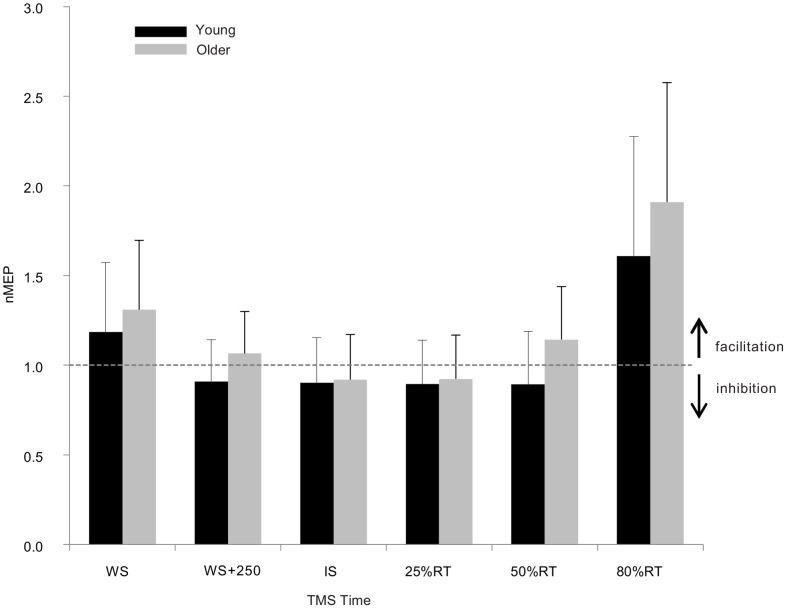
MEP sizes in response to single pulse stimulations (i.e., unconditioned responses) applied to right motor cortex during movement preparation/execution. Values are normalised to excitability at rest (dotted line) and shown for the young and older groups. Error bars represent 95% CIs.

Corticospinal excitability of projections to the L FDI at rest did not differ significantly between the two groups (1.25±0.32 mV and 1.31±0.64 mV for the young and older adults, respectively; independent samples *t*-test *t*_24 = _0.37, p = 0.713). Corticospinal excitability at the various time points within the movement preparation period were compared to the excitability observed at rest using nMEP (see Methods – *Data Analysis*). ANOVA revealed a non-significant effect of age (F_1,24 = _0.53; η_p_^2^ = 0.02; p = 0.472), and a significant effect of time (F_5,120 = _13.2; η_p_^2^ = 0.35; p<0.001). Figure 2 indicates that any changes in excitability were minimal early during preparation, but that a relatively large increase in excitability was observed just prior to onset of the response. The interaction of time and age was not significant, indicating the time-course of nMEP modulation was not dissimilar for the two age groups (F_1,24 = _0.40; η_p_^2^ = 0.02; p = 0.849).

### Interhemispheric connectivity

**Figure 3:**
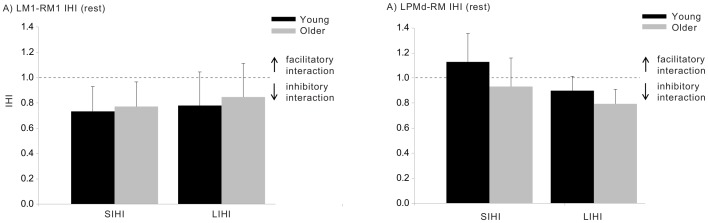
Interhemispheric interactions between LM1-RM1 (A) and LPMd-RM1 (B) for the young and older groups recorded at rest. Values <1 (horizontal dotted line) represent inhibitory interactions, while values >1 represent facilitatory interactions. Data are shown for the short and long ISIs and for both participant groups. Error bars represent 95% CIs.

LM1-RM1 and LPMd-RM1 IHI ratios, assessed at rest, are presented in figure 3. For LM1-RM1, the main effects of ISI (F_1,24 = _0.86; η_p_^2^ = 0.04; p = 0.362) and age (F_1,24 = _0.53; η_p_^2^ = 0.01; p = 0.724), and the interaction between ISI and age (F_1,24 = _0.06; η_p_^2^<0.01; p = 0.815) were all non-significant. As Figure 3A indicates, both age groups exhibited qualitatively similar interactions (i.e., IHI<1) at both 10 ms ISI (SIHI) and 40 ms ISI (LIHI). In contrast, for the LPMd-RM1 interactions, ANOVA revealed a significant effect of ISI (F_1,24 = _4.55; η_p_^2^ = 0.16; p = 0.043), with substantial inhibition exhibited at the 40 ms ISI (IHI = 0.85), but not at the short 8 ms ISI (IHI = 1.03) (figure 3B). The main effect of age (F_1,24 = _2.87; η_p_^2^ = 0.11; p = 0.103) and the interaction between age and ISI (F_1,24 = _0.28; η_p_^2^ = 0.01; p = 0.602) were not statistically significant.

**Figure 4:**
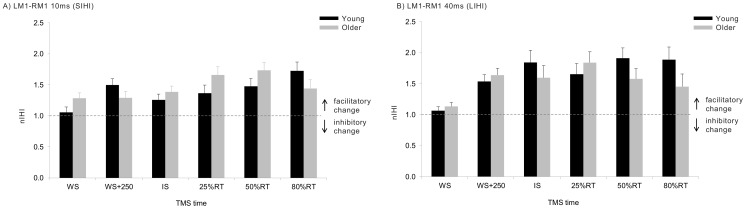
Modulation of LM1-RM1 interactions as a function of time. IHI values are shown for the 10 ms (A) and 40 ms (B) ISIs, and for both age groups, normalised to IHI expressed at rest. Values >1 (horizontal dotted line) represent a facilitatory change in the interation, relative to rest. Error bars indicate 95% CIs.

We next considered changes in LM1-RM1 and LPMd-RM1 interactions during task performance, relative to those interactions expressed at rest using nIHI (see Methods – *Data Analysis*; also [7]). For the LM1-RM1 pathway, the main effect of time was significant (F_5,120 = _3.69; η_p_^2^ = 0.13; p = 0.004), with the greatest release of inhibition relative occurring at 25%RT and 50%RT (figure 4). Indeed, from WS+250 ms onwards, the inhibitory interaction observed at rest (IHI<1, figure 3) had become a facilitatory interaction (IHI>1) for both SIHI and LIHI. The effects of ISI (F_1,24 = _3.69; η_p_^2^ = 0.03; p = 0.363, age (F_1,24 = _0.01; η_p_^2^<0.01; p = 0.932) and all two- and three-way interactions (all p>0.186) were not significant. Accordingly, the observed release of inhibition in the LM1-RM1 interaction as a function of time was not dissimilar for both SIHI (10 ms ISI) and LIHI (40 ms ISI), and both age groups.

**Figure 5:**
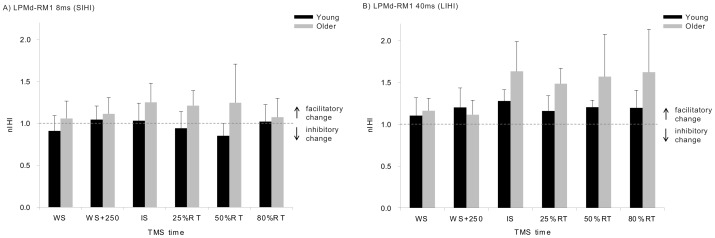
Modulation of the LPMd-RM1 interaction as a function of time. IHI values are shown for the 8 ms (A) and 40 ms (B) ISIsand for both age groups normalised to IHI expressed at rest. Values >1 (horizontal dotted line) represent facilitatory changes in the interation, relative to rest. Error bars indicate 95% CIs.

Figure 5 illustrates the nature of the PMd-M1 interactions during the movement preparation period (relative to rest) for both ISIs and both age groups. ANOVA revealed a significant main effect of age (F_1,24 = _5.07; η_p_^2^ = 0.17; p = 0.034), with the older group exhibiting greater modulation of the PMd-M1 interaction compared to the young group. The main effect of ISI was also significant (F_1,24 = _16.4; η_p_^2^ = 0.41; p<0.001). For SIHI (8 ms ISI) nIHI was 1.06: the inhibitory interaction at rest (figure 3) was marginally released but remained inhibitory. In contrast, for LIHI (40 ms ISI) nIHI was 1.31: relative to rest (where there was no significant inhibition, figure 3) the interaction became substantially facilitatory. The main effect of time was not significant (F_5,120 = _1.48; η_p_^2^ = 0.06; p = 0.201). The interaction between ISI and time (F_5,120 = _2.24; η_p_^2^ = 0.09; p = 0.054) just failed to reach significance, but indicates a strong trend for facilitatory changes in the PMd-M1 interaction late in the preparation period to be more pronounced for the LIHI mechanism (40 ms ISI) compared to those (minimal) changes observed for SIHI (8 ms ISI) (figure 5). All other interactions did not reach statistical significance (all p>0.168).

### Regression analyses

**Figure 6:**
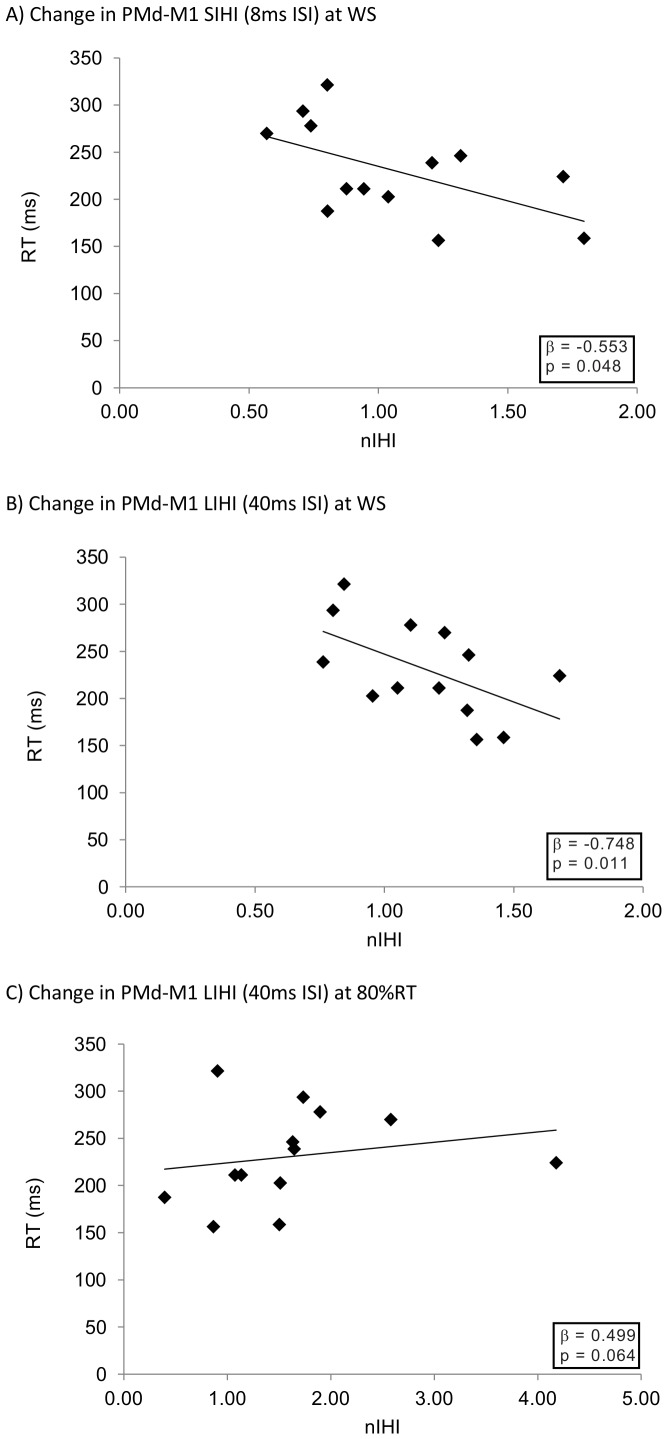
Associations between the individual predictors in the multiple regression model and response time. For PMd-M1 interactions, release of inhibition early in preparation (coincident with WS) was associated with faster responses for both 8 ms (panel A) and 40 ms (panel B). A very late release of PMd-M1 inhibition (at 80%RT) was weakly associated with slower response times, but only for 40 ms ISI (panel C).

Using multiple regression procedures we investigated whether the task-related modulation of IHI observed during movement preparation was associated with better task performance (i.e., faster RTs). For older adults, the specifically-timed modulation of short (8 ms) ISI LPMd-RM1 IHI (SIHI) at the onset of the WS predicted RT (model summary: R = 0.55, R^2^ = 0.31; F = 4.85, p = 0.045; β = −0.55): facilitation of the PMd-M1 SIHI interaction (which at rest did not exhibit significant inhibition or facilitation– figure 3) was associated with faster RTs in these older adults. For older adults, two predictors were included in the regression model linking the modulation of long (40 ms) ISI LPMd-RM1 IHI (LIHI) with RTs: in this instance, early (onset of WS) release of the inhibition that was observed at rest was associated with *faster* reaction but an inhibitory change (increase in inhibition) at 80% RT (i.e., just prior to movement execution) resulted in *slower* RTs (model summary: R = 0.72, R^2^ = 0.52; F = 5.33, p = 0.027; β = −0.75, 0.50 for early and late IHI modulation, respectively). For young adults, all predictors (independent variables) were excluded from the regression models for PMd-M1 pathways (at both ISIs) indicating that the modulation of PMd-M1 interactions did not adequately predict RT. Furthermore, regression models using LM1-RM1 interactions (at both ISIs) as independent variables failed to identify any significant predictors for reaction time for both young, and older, adults. The associations between the neurophysiological predictors of performance as derived in the multiple regression analyses, and the performance measure (i.e., RT) can be observed in figure 6. It is apparent that strong relationships exist between early modulation of PMd-M1 interactions for both SIHI and LIHI (8 ms and 40 ms ISIs - figure 6a, b), while the relationship between late PMd-M1 interactions and RT is far less pronounced (figure 6c) and but is included in the model by virtue of having a F probability of less than 0.10 (the exclusion criteria for the model predictors, see methods [6])

Overall, the regression analyses indicate that early facilitatory changes in both SIHI (from IHI∼1 into facilitation) and LIHI (reduction in the extent of inhibition observed at rest) between LPMd and RM1 (measured at 8 and 40 ms ISIs, respectively) are associated with faster reaction times in older adults highlighting the potential importance of LPMd in this task for older adults; a late facilitatory change in the LIHI LPMd-RM1 pathway (measured at 40 ms) was linked to slower reaction times for the older adults, although this relationship appears less robust.

## Discussion

While providing substantial insights into how the interactions between contralateral regions within the motor network are altered during movement preparation, existing studies [4]–[6] have focused predominantly on healthy young individuals. Given that older adults are thought be more reliant on cognitive strategies during motor tasks [32]–[34], and may activate different brain regions [47] - specifically premotor and frontal regions - findings in young adults may not necessarily be applicable to an older population. The present study specifically addressed this issue by comparing task-related modulation of interhemispheric interactions in groups of young and older adults. Interhemispheric interactions between contralateral primary motor areas (LM1-RM1) and between left premotor cortex (LPMd) and RM1 were investigated during a simple reaction time task. By using a simple reaction time task we were able to assess task-related changes in these interactions *in-situ* (c.f. [6]) and address the important question as to whether these neurophysiological measures can be used to predict motor performance for both young and older adults [27] – i.e., whether changes in these interhemispheric interactions represent task-specific functional connectivity between distinct brain regions.

At rest, young and older adults exhibited a similar degree of inhibition between the left and right primary motor cortices (figure 3a). This inhibition was apparent when paired pulse TMS was administered at both 10 and 40 ms interstimulus intervals to assess the purported SIHI and LIHI mechanisms. During movement preparation, these inhibitory interactions became more facilitatory (figure 4), a finding which is consistent with previous findings [4] and which supports the proposition that inhibitory interactions from the non-responding to the responding primary motor cortex become less inhibitory to release the impending action. This ‘release’ was most pronounced from the onset of the imperative signal onwards, and did not vary significantly between the young and older groups. Previous reports indicate that older adults may exhibit diminished modulation of IHI assessed at 40 ms ISI, [26], but not at 10 ms ISI [7], [26], during a task requiring accurate upper limb control to produce specific forces (force matching task). It may be the case, therefore, that precise tasks requiring feedback control to achieve the goal outcome (compared with ballistic actions), accentuate age-differences in interhemispheric control mechanisms, particularly LIHI. Chen and colleagues [22], [46] have suggested that measuring M1-M1 IHI at short ISIs (i.e., SIHI) may probe direct pathways, while using a longer ISI (i.e., LIHI) may assess indirect pathways, conceivably involving premotor regions. Furthermore, LIHI may be mediated by postsynaptic GABA_B_ receptors [22], [23] whereas at present it is unclear which mechanisms mediate SIHI. Accordingly, the previously observed age-related degradation in the ability to modify M1-M1 LIHI during a motor task may actually be a result of age-related decline in *premotor* functionality or reduction in the efficacy of GABA_B_ receptors. In the present task, however, participants were not required to accurately attain specific force levels, but simply produce a fast-as-possible response. Furthermore, in the present task we utilised a warning signal with the aim of promoting movement preparation (relative to a task without a warning signal). These task differences may have resulted in our finding that older adults were able to modulate both M1-M1 SIHI and LIHI to a similar extent to the young group.

Interhemispheric interactions were also assessed between left premotor cortex and right primary motor cortex. Previous studies have generally assessed premotor-motor interhemispheric interactions using an ISI of between 2–15 ms [15], [16], [48] to measure SIHI, although it has recently been shown that LIHI can be observed in PMd-M1 interactions [25]. Here, for the first time, we assessed how both SIHI and LIHI mechanisms between LPMd and RM1 are modulated during movement preparation/execution, and how this may be affected by ageing. At rest, the influence of LPMd conditioning on RM1 cortical output was less pronounced compared to that conditioning effect observed as a result of LM1 conditioning (figure 3b). LPMd conditioning did not reliably affect the amplitude of the MEP evoked in the left FDI at short (8 ms) ISIs, a finding which is consistent with recent observations [45]. There was, however, evidence of inhibitory interactions at the longer (40 ms) ISI, evidenced by a IHI ratio of 0.85 averaged across both age groups (c.f. [25]). As with M1-M1 interactions, there were no differences in PMd-M1 IHI ratios at rest (for SIHI or LIHI) between the young and older groups.

A novel finding of the present experiment was that we observed significant age-differences in the task-related modulation of LPMd-RM1 interactions. As shown in figure 5, and supported by statistical analysis, the older group exhibited a larger degree of modulation of LPMd-RM1 interactions during movement preparation compared to the young adults. Specifically, only for older adults, and most noticeably at the longer ISI (i.e., LIHI), significant facilitatory changes (the inhibitory interaction at rest switch into facilitation) were observed (group averaged data) from the onset of the imperative signal, and remained facilitatory for the remainder of the preparation period (figure 5B). For the first time, we have shown task-related facilitatory changes in LIHI between PMd and contralateral M1, which were more pronounced than the task-related changes in SIHI. This finding supports the hypothesis that short (8 ms) and long (40 ms) ISIs assess two distinct transcallosal pathways/mechanisms [22], [25], [26], [46].

To determine if task-related modulation of interhemispheric interactions was ‘functional’ with respect to speeding reaction times, we undertook multiple regression analyses [6]. The substantial modulation of LM1-RM1 interactions that was observed for both age groups (figure 4) did not predict reaction times for either age group. It is conceivable that release of M1-M1 IHI is functionally-related to some other aspect of the task, for example peak movement speed or peak acceleration of the finger during the ballistic movement. We observed that an early (but not late) facilitatory change in LPMd-RM1 interactions was associated with faster reaction times. This was only true, however, for *older adults*. Specifically, for older adults, more facilitatory influence from LPMd onto RM1 (i.e., the responding primary motor cortex) at the time of the warning signal, was associated with the fastest reaction times. This correlation was observed for both short (8 ms) and long (40 ms) ISIs (SIHI and LIHI, respectively), suggesting that even though task related modulation of PMd-M1 LIHI was more pronounced than modulation of SIHI, task-related changes in both SIHI and LIHI correlated with behaviour. This finding is consistent with the hypothesis that early modulation of interactions emanating from LPMd plays a dominant role during movement preparation [11]–[13]. In addition, we observed that, for older adults, the modulation of the LPMd-RM1 LIHI late during movement execution (observed as more facilitatory connections relative to rest, figure 5b) was actually associated with a *slowing* of reaction times. This association is unexpected, especially as the time point at which this correlation was observed (80% RT) represents a point so late in movement execution (immediately prior to onset of muscle activity) that the LPMd would not, given extant theories, be expected to play a critical role. This particular correlation was associated with a lower magnitude β value (0.50) compared to the correlation associated with early PMd-M1 LIHI modulation (−0.75), and a weaker correlation when plotted against reaction time (figure 6c), reiterating that the aforementioned *early* modulation of PMd-M1 interactions are strongest, and likely the most task-relevant, associations. Indeed, it may be that this weaker association between nIHI at 80%RT and reaction speed is an artefact resulting from the large excitability increase [49] at this late time point (figure 2), or driven by one or two participants with particularly high nIHI values (figure 6c).

The current data indicate that, for older adults, an ability to regulate LPMd-RM1 interhemispheric interactions *early* during movement preparation is paramount in permitting fast reactions to external cues and represents functional connectivity between these distinct interhemispheric regions. The fact that young adults did not exhibit substantial modulation of LPMd-RM1 interactions during movement preparation and did not show significant correlations between interhemispheric interactions and performance may suggest that young adults did not rely on premotor regions during this simple task to the same extent as the older adults. Our data do not permit us to state that age-related changes in LPMd-RM1 interactions as the sole influencing factor, and assessing causality through virtual lesion studies (e.g. theta burst stimulation or double pulse TMS) may shed further light on this issue. As figure 5 reveals, *averaged over all older participants*, only modest facilitatory changes were observed in the LPMd-RM1 interaction at the onset of WS (at either ISI). However, for the older group nIHI at WS onset ranged between 0.79–1.79 (8 ms ISI - SIHI) and 0.76–1.46 (40 ms ISI - LIHI); accordingly, those participants who exhibited the greatest facilitatory change in the interaction (relative to rest, i.e., highest nIHI values) very early in the preparation phase exhibited the fastest reaction times. Because this facilitation occurred coincident with WS, it appears that a number of participants were able to display a rather ‘generalised’ task-related facilitatory change in the interhemispheric interaction, which was apparent before neural responses to the WS could have occurred. Such early preparatory changes are conceivable, given that in this particular task, the required response was highly predictable: over 90% of all trials required a response (only 3 trials out of 36 in each block were ‘catch’ trials) and this response was always with the left hand. Importantly, we note that no association between IHI ratios at rest and response times were observed lending weight to the proposition that only early *task-related modulation of PMd-M1interactions* were associated with faster responses.

In the present study as well as assessing task-related changes in interhemispheric interactions, single pulses of TMS were used to assess excitability of the corticospinal projections to the left FDI, the muscle primarily involved in task execution. Older adults exhibited excitability changes in the movement preparation period that were indistinguishable from the young adults. Specifically, for both groups a small, statistically non-significant, suppression of excitability was seen early in the preparation period, followed by facilitation immediately prior to movement execution (at 80% RT, figure 2). Suppression of corticospinal excitability in the early stages of movement preparation has been reported previously [50]–[53], and is thought to prevent early release of the motor action [52]. In the present task, increases of excitability just prior to EMG (response) onset were not disimilar for young and older adults, supporting the proposition that older adults are able to prepare a planned action as well as young adults, at least when the required response is predictable (i.e., not a choice reaction time, or Go-NoGo task, for example - [54]). It has been argued that MEP sizes in response to single pulses of TMS should be normalised across conditions/time points such that subsequent measurement of IHI is unaffected by changes in excitability (e.g. [49], but also see [20] and [19]). However, it is noteworthy to mention that because no age-related differences were observed in excitability at any time point, the significant age-related changes in the modulation of LPMd-RM1 interactions in the present study cannot simply be explained by excitability changes, and therefore likely represent an independent, task-related, preparatory mechanism. While we acknowledge that the large release of M1-M1 inhibition observed at 80% RT for both age groups (figure 4) may be due, at least in part, to the increase in excitability at this time point, earlier releases of M1-M1 inhibition (at WS+250 ms and 50%RT, figure 4) which were equally prominent occurred when *no* change in excitability was observed. As such, we suggest the observed changes represent task-specific modulation of interhemispheric interactions, rather than being driven by excitability change.

Finally, we note that changes in MEP size in the *right* hand in response to the conditioning stimuli applied to LM1 did not vary between age groups, nor did they change as a function of time during movement preparation/execution relative to rest. Accordingly, excitability of the ‘conditioned’ hemisphere was not significantly affected during task preparation/execution. Conditioning stimuli applied to LPMd did not lead to recordable responses (MEPs) in the right FDI, lending weight to the argument that the reported changes in LPMd-RM1 interactions were primarily due to LPMd stimulation, and were not due to erroneous LM1 stimulation as a consequence of ‘spreading’ of the stimulation. In summary, this study revealed differences in the manner that adults of varying ages modulate interhemispheric interactions during a simple reaction time task. The present findings extend our understanding of the role of left premotor cortex during planning and execution of ballistic motor tasks, by providing evidence of functional connectivity between left premotor cortex and right primary motor cortex during task performance in older age. This builds on previous findings indicating functional modulation of PMd-M1 interactions for movement control, for example during bimanual coordination [6] and, in a broader context, adds to the literature indicating functional modulation of interhemispheric interactions emanating from PMd, for example correlations with performance during tactile perception [55]. The current data are consistent with the view that ageing may be associated with a greater reliance on premotor regions in simple motor tasks. Furthermore, we have shown that investigating short and long interval IHI [25] between premotor and primary motor regions during motor preparation may be particularly useful in ageing research as these different mechanisms may be differentially affected by age [26]. Further work is warranted to investigate the specific timing of task-related changes in interhemispheric interactions between primary and premotor regions, and how this may be related with different parameters of task performance across different movement tasks and across various age groups. Studies which aim to determine *causal* relationships between neurophysiological function (in this case, IHI) and motor performance appear critical if this knowledge can be applied in clinical contexts with the aim of improving motor function in older age, and recovery following brain injury (e.g. stroke) or periods of limb immobilisation due to injury [27].
